
JOURNAL INFORMATION
==============================
NLM Title Abbreviation: Halls J Health
NLM Journal ID: 0011644

Hall's Journal of Health

Publisher: Biomedical Journal Digitization Project

ARTICLE INFORMATION
==============================
PMCID: PMC9192682
Article version: 1
Subjects: Articles

Notices

Print publication date: 1869 Nov
Volume: 16
Issue: 11
First page: 290
Last page: 292
Page range: 290, 292
License: This work is free of known copyright restrictions. For more information, please see PMC Back Issue Digitization.
License URL: https://creativecommons.org/publicdomain/mark/1.0/

==============================

### NOTICES.

INVALIDS who may require a warmer latitude for the winter will find at Nassau, New Providence, a home at the Royal Victoria Hotel under American management. It is the capital of the Bahama Islands, twenty-three miles long and seven broad. During the winter months the thermometer varies but about eight degrees; the average temperature from Nov. 1st to May 1st being about seventy-two degrees Farenheit. References are given by the proprietors, Cleveland and Newell, to two of our most distinguished surgeons, Prof. Flint of Thirty-fourth Street, and Dr. James R. Wood.

BOYS CLOTHING of every style furnished by Baldwin the clothier at north-east corner of Broadway and Canal Street at fair prices, well made and of honest material. All are dealt fairly by, hence their large business.

THE AMERICAN INSTITUTE holds its thirty-eighth annual exhibition for 1869 at the Rink on Third Avenue and Sixty-third Street, New York. The importance, interest and success of the present exhibition may be inferred from the fact, that two thousand dollars are received in a day from persons who desire to witness this extraordinary display of human ingenuity, skill and power.

---

THE CELEBRATED

**PATENT CRAIG MICROSCOPE**

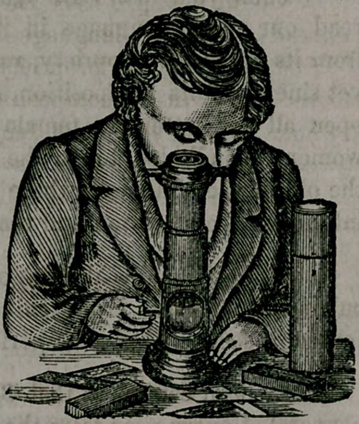

Magnifies ten thousand times — a power equal to other microscopes of many times its cost. Reveals the globules in milk, blood, and other fluids, solids or salts of urine, animalcules and minerals in water, cancer cells, eels in vinegar, claws and hairs of insects, shape of the pollen of flowers, hundreds of eyes in the single eye of a fly, tubular structure of hair, dust of a butterfly's wing to be perfectly formed feathers, adulterations of food, and also the celebrated *trichina spiralis* or pork-worm, which has caused so much excitement in this country, and which was *first* discovered here with *this* microscope by Dr. R. C. Kendall, of Philadelphia. Price only $2.50. Mailed, post-paid, for $2.75.

Address the sole proprietor,

GEORGE MEAD, Chicago, Ill. Box 1085, P.O.

Also for sale on same terms at the office of "Hall's Journal of Health," 176 Broadway, N. Y.

WM. WOOD & CO. have published "ANAL FISSURE, its Aetiology, Pathology, Diagnosis and Treatment," by William Bodenhamer, A.M. M.D., author of Congenital Diseases of the Rectum, and Professor of Diseases of the Rectum and Contiguous Textures, 199 pp. 8vo., illustrated by numerous cases and drawings. The editor, after a personal acquaintance with the Author of more than thirty years, and a familiarity with his mode of practice, does not hesitate to pronounce Dr. Bodenhamer as the most successful living practitioner at home or abroad in ANAL FISSURE, FISTULA, STRICTURE of the bowels, PILES.

His manipulations performed in a manner peculiar to himself, are shown and communicated to the Profession with great courtesy and cheerfulness when called upon at his residence, 237 5th Ave., New York; he has not only obtained its confidence and respect here, but has drawn to himself an amount of practice from a list of patients of which he may well be proud, members of various state legislatures, governors, senators, officers in the Army and Navy, and from all the professions, and we feel that we are doing our readers a very great service in making these statements, (without the author's knowledge) in case any of them should need his skill in the direction referred to.

As the cases given are very instructive to the general reader, and the causes of these most distressing diseases are plainly stated, and by knowing them the maladies may be avoided, we will keep the book for sale in our office, or will send it post-paid on having $2.25 remitted in a post office order, for safety, to address of HALL'S JOURNAL OF HEALTH, 176 Broadway, New York.
